# Tropical influenza and weather variability among children in an urban low-income population in Bangladesh

**DOI:** 10.3402/gha.v7.24413

**Published:** 2014-08-12

**Authors:** Chisato Imai, W. Abdullah Brooks, Yeonseung Chung, Doli Goswami, Bilkis Ara Anjali, Ashraf Dewan, Ho Kim, Masahiro Hashizume

**Affiliations:** 1 Department of Pediatric Infectious Diseases, Institute of Tropical Medicine, Nagasaki University, Nagasaki, Japan; 2 Graduate School of Biomedical Sciences, Nagasaki University, Nagasaki, Japan; 3 Japan Society of the Promotion of Science, Tokyo, Japan; 4 International Centre for Diarrhoeal Disease Research, Bangladesh (ICDDR, B), Dhaka, Bangladesh; 5 Johns Hopkins Bloomberg School of Public Health, Baltimore, MD, USA; 6 Department of Mathematical Sciences, Korea Advanced Institute of Science & Technology, Daejeon, South Korea; 7 Department of Spatial Sciences, Curtin University, Bentley, WA, Australia; 8 Department of Geography & Environment, University of Dhaka, Dhaka, Bangladesh; 9 Department of Biostatistics and Epidemiology, Graduate School of Public Health & Institute of Health and Environment, Seoul National University, Seoul, South Korea

**Keywords:** influenza, tropics, weather, low-income, poor, urban, children, time series

## Abstract

**Background:**

Influenza seasonality in the tropics is poorly understood and not as well documented as in temperate regions. In addition, low-income populations are considered highly vulnerable to such acute respiratory disease, owing to limited resources and overcrowding. Nonetheless, little is known about their actual disease burden for lack of data. We therefore investigated associations between tropical influenza incidence and weather variability among children under five in a poor urban area of Dhaka, Bangladesh.

**Design:**

Acute respiratory illness data were obtained from a population-based respiratory and febrile illness surveillance dataset of Kamalapur, a low-income urban area in southeast Dhaka. Analyzed data were from January 2005 through December 2008. Nasopharyngeal wash specimens were collected from every fifth eligible surveillance participant during clinic visits to identify influenza virus infection with viral culture and reverse transcriptase–polymerase chain reaction. Time series analysis was conducted to determine associations between the number of influenza cases per week and weather factors. Zero-inflated Poisson and generalized linear Poisson models were used in the analysis for influenza A and B, respectively.

**Results:**

Influenza A had associations with minimum temperature, relative humidity (RH), sunlight duration, and rainfall, whereas only RH was associated with influenza B. Although associations of the other weather factors varied between the two subtypes, RH shared a similar positive association when humidity was approximately 50–70%.

**Conclusions:**

Our findings of a positive RH association is consistent with prior studies, and may suggest the viral response in the tropics. The characteristics of settlement areas, population demographics, and typical overcrowding of urban poverty may also contribute to different impacts of rainfall from higher economic population. Further investigations of associations between tropical influenza and weather variability for urban low-income populations are required for better understanding.

Influenza is one of the most significant diseases in humans, given its high morbidity and mortality worldwide (1). Epidemics of seasonal influenza impose substantial health burdens on all age groups in the population, with the highest risk of developing influenza-related complications primarily among children younger than age 5 and elderly aged 65 or older (2). Although influenza has long been associated with high hospital admissions and mortality among the elderly, its impact on young children has recently drawn increased attention because of its significant instances of childhood pneumonia morbidity and mortality (3–5). Given that pneumonia is the leading cause of death in children under five in the world (6), controlling influenza epidemics is important for childhood morbidity and mortality reduction.

To date, yearly influenza outbreaks in temperate climate regions have been distinctively observed during wintertime in both the Northern and Southern Hemispheres, and the linkage between influenza and dry-cold weather is widely acknowledged (7). The correlation between influenza and weather variability in fact corresponds to mechanisms of the air-borne survival of lipid-enveloped influenza viruses, which explains its greater survival at lower temperature and lower relative humidity, or its lower survival at higher temperature and higher humidity (8). However, this mechanism alone does not fully explain seasonality characteristics in tropical regions. Influenza infections in the tropics are often reported throughout the year, and major peaks are usually observed during rainy seasons, when humidity and temperature are high (9–11). Reasons for this seasonality difference between temperate and tropical climate regions remain largely unknown, partially because the seasonality in the tropics has been less documented and poorly understood.

In earlier studies, underlying mechanisms of influenza seasonality have been potentially linked, not only to temperature and humidity but also other factors, such as vitamin D from supplement intakes and exposure to solar radiation (12, 13), geographic latitude (14, 15), and indoor crowding by rainfall or at schools (16, 17). Given these findings, we focused on associations of tropical influenza with four weather factors, namely, temperature, relative humidity, sunlight duration, and rainfall. Children in an urban low-income population were targeted as the study population. The urban population continues to rise, representing more than half of the global population, and nearly one third of the urban population consists of slum dwellers (18, 19). Inadequate sanitation, limited resources, and high levels of overcrowding that characterize conditions of urban poverty are known to make the impoverished residents highly vulnerable to communicable diseases such as acute respiratory diseases (20–22). There are public health concerns with the growing urbanization and low-income population, yet little is known about health burdens on the urban poor because of limited data (21). In response to this lack, tropical influenza and weather variability are investigated for the target population in Bangladesh.

## Methods

### Data

In 1998, the International Center for Diarrheal Disease Research, Bangladesh (ICDDR, B) established a population-based surveillance system in Kamalapur, a poor area of Dhaka, Bangladesh. The surveillance design and characteristics have been described elsewhere (5, 23). Briefly, children younger than age five living in Kamalapur were selected by stratified cluster random sampling and followed longitudinally during weekly home visits by trained field research assistants (FRAs). The FRAs used standardized questionnaires to elicit both recall and observational data on clinical illness signs each day of the week since their previous visits. Major signs included fever, tachypnea, chest indrawing, lethargy, cyanosis, inability to drink, and convulsions. Minor signs included cough, rhinorrhea, sore throat, myalgia/arthralgia, chills, headache, irritability/decreased activity, and vomiting. The FRAs referred children with one major or at least two minor signs to an onsite clinic. All clinical evaluations were conducted free of charge.

In the clinic, project physicians and nurses conducted standardized clinical examinations to make standardized diagnoses of febrile or respiratory illnesses. Nasopharyngeal wash (NPW) specimens were collected from every fifth child (20%) with either an axillary temperature (≥38°C) or elevated respiratory rate (≥60/min if <60 days of age, ≥50/min if 60–365 days old, and ≥40/min if 1–5 years old) and one of the following clinical signs localized the disease to the respiratory tract: cough, chest indrawing, inspiratory crepitations, expiratory wheezes, or rhonchi. The NPW was then tested by viral culture through May 2008 and with reverse transcriptase–polymerase chain reactions (RT–PCRs) thereafter to identify pathogens. Data from April 1, 2004, through March 31, 2011, for the Kamalapur surveillance were initially obtained from ICDDR, B. However, data before 2005 and after 2008 were discarded from the analysis because a large portion of the 2004 data were missing because of severe flooding, and after 2009 seasonality was distorted by the emergence of the novel influenza H1N1 virus. Consequently, 4 years, from January 1, 2005, through December 31, 2008, were used as the study period. Daily minimum and maximum temperatures (°C), relative humidity (RH, in%), sunlight duration (hour), and rainfall (mm) in Dhaka were collected from the Bangladesh Meteorological Department.

### Statistical analysis

A time series method was applied to investigate associations between influenza and weather variability. Daily numbers of laboratory-confirmed cases of influenza A and B were aggregated by week and considered as dependent variables. As independent variables, weekly averages of minimum temperature, RH, sunlight duration, and total weekly rainfall were included in multivariate models. Associations of those factors with influenza were found in prior studies (13–16). Influenza B was analyzed with a generalized linear Poisson regression model. A zero-inflated Poisson regression model was used for influenza A, because there were an excessive number of weeks with no confirmed infection cases. For non-linearly associated weather factors, natural cubic splines were first used in the models, and then piecewise linear regressions were applied to estimate break points and effects below and above those points. Optimal break points were determined by the lowest maximum likelihood estimation through iterative fitting of the threshold points and linear models (24). For analyzing delayed effects of weather factors, long- and short-lag effects were defined by a moving average of the current and previous week (0–1 week average), and of the current week through 3 weeks prior (0–3 week average). Each subtype model was thereby further developed into two different lag models. Other than weather factors, seasonal variations were controlled in the models by including year and season variables (January–March, April–June, July–September, and October–December) for influenza A and a month variable for influenza B. As a confounder, an indicator for different laboratory methods (viral culture and RT-PCR) was also fit in the models to adjust for sensitivity of detecting the pathogen. Finally, at the end of building models, one week-lagged residual errors were included in the models as a predictor to control remaining autocorrelations (25). The statistical analyses were performed with the statistical package R version 2.15.3 (26).

### Sensitivity analyses

Prior to fitting the zero-inflated Poisson model, model fitness of the generalized linear negative binomial and zero-inflated negative binomial were compared by Vuong’s test and likelihood ratio test (27). The optimal degrees of freedom for natural cubic spline terms of non-linear meteorological variables were selected based on the lowest Akaike’s Information Criterion (AIC). As alternative methods for seasonal controls, periodic harmonic functions called Fourier series, formed by the sum of sines and cosines, and smoothing splines of observation time were also fit in the models, and compared by AIC, deviance, and overdispersion parameters. Data on ambient temperature were available for both minimum and maximum temperatures. These two parameters did not exhibit a significant difference in the goodness of fit of the models. Therefore, minimum temperature was selected for its slight improvement of AIC and deviance, and for its temperature range comparable with preceding studies, which were mostly done in temperate climates.

## Results

**Fig. 1:**
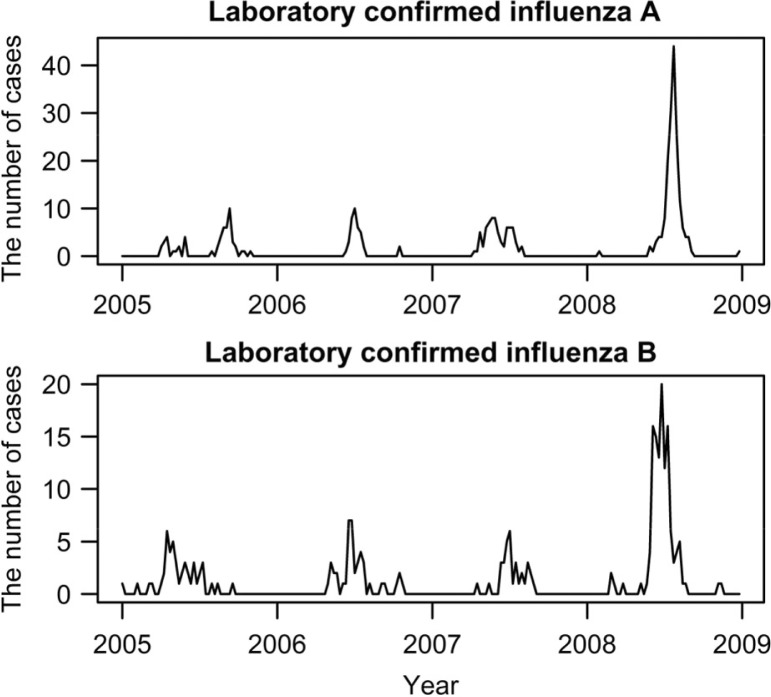
Time series plots for laboratory confirmed influenza A and B per week in Kamalapur, 2005–2008.

**Fig. 2:**
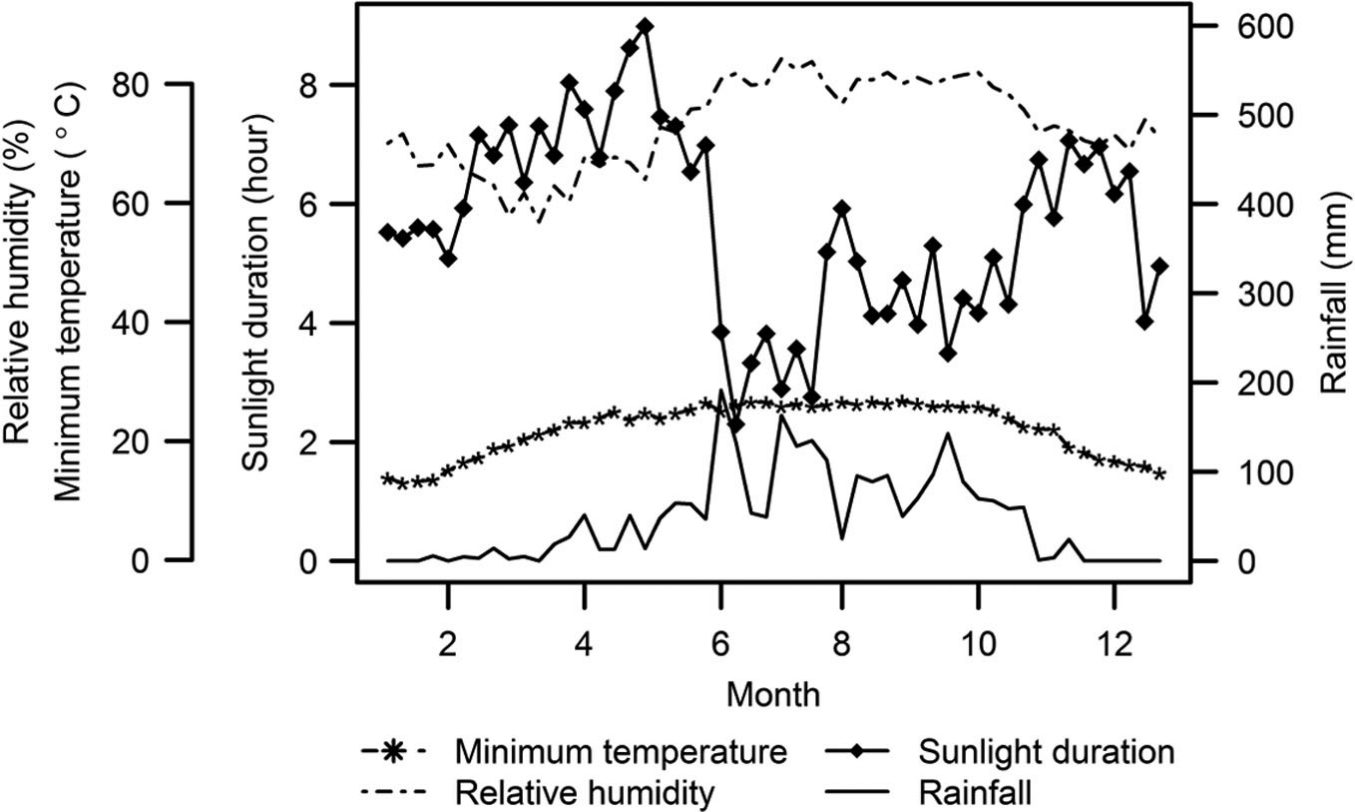
Weekly average of minimum temperature, relative humidity, sunlight duration, and rainfall in Kamalapur, 2005–2008.

During the study period, there were 14,140 clinic visits and 3,198 NPWs collected. Among the collected specimens, the number of confirmed infections with influenza A and B virus were 333 and 246, respectively. Figure 1 shows the weekly number of cases during the study period. One distinctive peak was observed in each subtype every year, during May through September. For the peaks in 2008, the number of cases drastically increased for both influenza subtypes. This is attributed to the excessive NPW collection rate and new laboratory method (RT-PCR), which replaced the less sensitive viral culture method from May 2008. These two factors were considered in the models to be adjusted. Table 1 shows summary statistics for weather variables. With a little variation, weekly minimum temperature and RH were high throughout the year, with averages of 22°C and 73%, respectively. The heaviest precipitation was from June through September, which was the period of highest temperature and RH as well as the shortest sunlight duration throughout the year (Fig. 2).

**Table 1:** Statistical summary for weekly average of minimum temperature, relative humidity, sunlight duration, and weekly total rainfall

Weather variables	Mean	S.D.	Minimum	Maximum
Minimum temperature (°C)	22.23	4.60	11.09	28.60
Relative humidity (%)	73.23	8.88	46.29	89.29
Sunlight duration (hour)	5.66	2.31	0.13	9.87
Rainfall (mm)	47.24	70.25	0.00	404.00

**Fig. 3:**
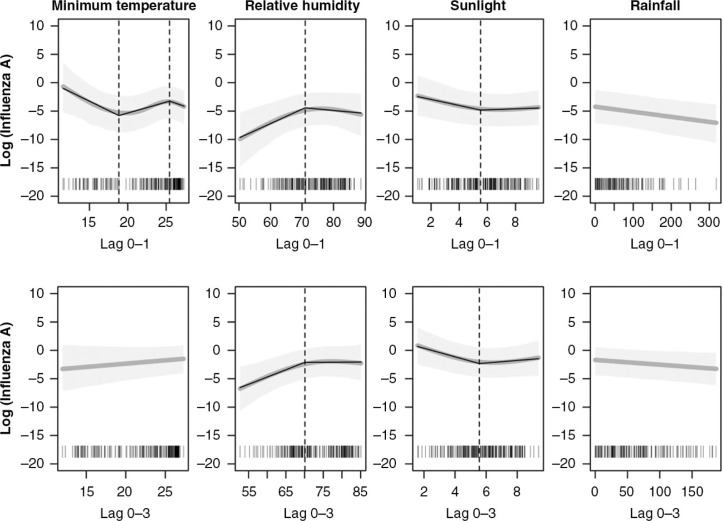
The upper and lower rows respectively represent influenza A at the moving average of week 0–1 and week 0–3. The estimates (dark gray line) and 95% confidence intervals (light gray shade) of adjusted predictions from the short-lag time models with natural cubic splines are presented. For non-linearly associated weather factors, piecewise regressions (black solid line) are additionally applied. The vertical dotted lines present the break points.

**Fig. 4:**
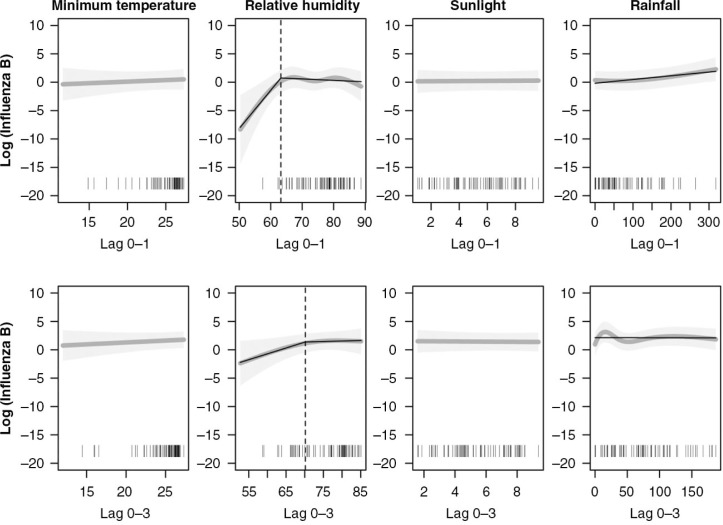
The upper and lower rows respectively represent influenza B at the moving average of week 0–1 and week 0–3. The estimates (dark gray line) and 95% confidence intervals (light gray shade) of adjusted predictions from the short-lag time models with natural cubic splines are presented. For non-linearly associated weather factors, piecewise regressions (black solid line) are additionally applied. The vertical dotted lines represent the break points.

Time series analysis revealed that the two influenza subtypes had different associations with weather variability, although association patterns between short (lag 0–1 weeks) and long (lag 0–3 weeks) time lags within each subtype were mostly similar. Figures 3 and 4 show estimates (dark gray line) and confidence intervals (light gray shade) of the associations between influenza and each weather variable from the short- and long-lag models. For the non-linear weather variables, estimated piecewise linear regressions (black solid line) and break points (black dotted line) are also presented in the figures.

Table 2 indicates that influenza A was significantly associated with RH, sunlight, and rainfall. A 1% increment of humidity in both short- and long-lag models significantly increased the risk of influenza A (27%) when humidity was approximately below the breakpoint, of 70% (*p*<0.01 for both subtypes). Because the lowest observed humidity in the study was 47%, the risk estimation only applies to RH from 47% through 70%. On the contrary, sunlight duration was negatively associated with influenza A, with approximately 37% risk reduction by 1 hour increase until the 5.5- to 5.6-hour break point in both the short- and long-lag models (*p*<0.01 for both). Rainfall also had a significant negative association, but the response was linear. Only temperature had a different response between the two different time lag models, with temperature-dependent associations in the short-lag model.

**Table 2:** Risk change in the number of influenza A per week for 1 unit increase in temperature (°C), relative humidity (%) and sunlight duration (hour) and for 10 mm increase in rainfall

		Risk Change (%)		
		
Weather variable	Break point	Estimate	95% CI	*P* d
0–1 week average lag				
Temperature (low)^a^	18.9	−51.03	(−73.49 to −10.63)	0.02*
Temperature (middle)^b^	25.5	60.16	(14.71 to 132.29)	<0.01*
Temperature (high)^c^	–	−43.39	(−66.35 to −3.03)	0.04*
Relative humidity (low)^a^	71.0	27.00	(6.57 to 51.65)	<0.01*
Relative humidity (high)^c^	–	−5.45	(−19.49 to 10.16)	0.47
Sunlight (low)^a^	5.5	−36.62	(−56.5 to −8.39)	0.01*
Sunlight (high)^c^	–	0.40	(−26.92 to 36.83)	0.98
Rainfall	–	−8.61	(−9.52 to −9.52)	<0.01*
0–3 week average lag				
Temperature	–	16.91	(−7.20 to 47.28)	0.19
Relative humidity (low)^a^	70.2	30.96	(8.77 to 57.67)	<0.01*
Relative humidity (high)^c^	–	0.93	(−22.92 to 32.16)	0.95
Sunlight (low)^a^	5.6	−43.77	(−58.72 to −23.41)	<0.01*
Sunlight (high)^c^	–	7.66	(−31.62 to 69.50)	0.75
Rainfall	–	−7.65	(−14.64 to −0.08)	0.05*

a The piecewise linear regression below the first break point.

b The piecewise linear regression above the first break point and below the second break point.

c The piecewise linear regression above the second break point.

d* for *P* value denotes<0.05.

Influenza B was less significantly associated with weather variability than influenza A. No evidence foran association between the number of influenza B cases and minimum temperature, sunlight duration, or rainfall was found for the short- and long-lag models (Table 3). The non-linear response of influenza B to RH, however, resembled that which appeared in the influenza A analysis. RH below the 63 and 70% break points in the respective short- and long-lag models had significant positive associations (*p=*0.02 and 0.05). RH above the break points did not have significant associations as observed with influenza A.

**Table 3:** Risk change in the number of influenza B per week for 1 unit increase in temperature (°C), relative humidity (%) and sunlight duration (hour) and for 10 mm increase in rainfall

		Risk Change (%)		
		
Weather variable	Break point	Estimate	95% CI	*P* c
0–1 week average lag				
Temperature	–	2.84	(−15.3 to 25.36)	0.77
Relative humidity (low)^a^	63.2	85.34	(9.50 to 215.57)	0.02*
Relative humidity (high)^b^	–	4.02	(−12.89 to 5.97)	0.40
Sunlight	–	2.08	(−17.83 to 16.93)	0.82
Rainfall	–	3.05	(0.00 to 0.00)	0.16
0–3 week average lag				
Temperature	–	9.53	(−6.46 to 27.99)	0.23
Relative humidity (low)^a^	70.3	13.43	(1.25 to 28.09)	0.05*
Relative humidity (high)^b^	–	5.44	(−8.35 to 20.59)	0.47
Sunlight	–	11.29	(−10.02 to 38.49)	0.34
Rainfall	–	−1.00	(0.00 to 0.00)	0.85

a The piecewise linear regression below the first break point.

b The piecewise linear regression above the second break point.

c* for *P* value denotes<0.05.

## Discussion

Multivariate analysis showed that associations between influenza and weather variability differed by influenza subtype. More weather factors were significantly associated with influenza A than influenza B. These findings are similar to those of other studies (28–30). The non-linear association of RH was, however, commonly observed with both subtypes, regardless of time lag. For the break point at ~70% RH, the risk appeared to significantly increase up to that point, then attenuated in excess of it. This RH-dependent result was previously documented in viral transmission studies in laboratory settings (31–33). Given that the lowest RH observed in the present study was 47%, it is limited to evaluating effects below ~50% RH. Nonetheless, the finding of a positive association for ~50–70% RH corresponds to results of other studies that examined wider ranges (20–80%) and found a U-shape association with RH. Those studies (31, 33) documented maximum transmission at low RH (≤40%), minimal at intermediate relative RH (50%), and high at elevated RH (60–80%). Such a bimodal relationship is considered a potential explanation for epidemics during the dry-cold season in temperate regions and during the humid-rainy season in tropical regions (15, 34). Our similar finding of increased risk between intermediate (50%) and high (70%) levels of RH supports and emphasizes this bimodal assumption.

In influenza A analysis, sunlight duration was another significant indicator for influenza incidence. Its negative association with influenza A appears compelling along with findings from other studies, and reinforces the underlying hypothesis that susceptible hosts might be protected by improved immunity in response to vitamin D photosynthesis induced by sunlight exposure (12, 13, 35). In contrary, the negative association of rainfall contradicts another population-based study that documented a positive association (15). The positive association of rainfall is often associated with the hypothesis that rainfall favors indoor crowding, thereby increasing direct-contact transmissions (7). A possible explanation for the discrepancy of results is that such a hypothesis is only valid for areas where the population is generally sparse, for instance, where people of higher economic status reside. That is because if crowding is indeed critical for the positive rain effect, where great overcrowding is common as in urban low-income communities, active transmissions should be routine. Thus, the seasonal variation of transmission cannot be explained by overcrowding in the urban poor population and should be attributed to other factors, such as RH as we found in the present study. However, this leads to another question, i.e. why is crowding not causing routine infections? The populations generally considered critical for containing transmission have different demographics in urban low-income communities. For instance, school-age children, the key population for premature immunity and close contact in schools (36), have a low rate of school attendance in Kamalapur as the consequence of poverty (37). According to our census database of Kamalapur, adults aged 65 or older, the other population in which there may be active transmissions, is likewise very small (<1% of the total population). Like many other low-income urban areas, this community consists predominantly of young working-age people who migrated from rural areas to seek jobs in the city. This population demographics may reduce impacts of crowding on children by providing them opportunities for immunity acquisition or herd immunity. Another unique characteristics of urban poverty may also be linked to the negative impact of rain. The urban poor often reside in ill-equipped sites or areas vulnerable to natural disasters (38). In particular, Kamalapur sits on low ground in Dhaka, a city reported to be in one of the world’s most flood-prone regions (39). Because a large portion of the area becomes inundated almost every year during monsoon season, the negative impact of rain perhaps resulted from temporary population displacement or disrupted transmissions, owing to inundation or flooding.

The association of temperature with influenza A is also counterintuitive. This appears to contradict the lipid-enveloped virus mechanism and other research findings (8, 40). However, one study in Hong Kong (29) showed a similar bimodal relationship, except for extreme temperature (≥26°C). These findings are perhaps attributable to the complexity of various factors of biology and behaviors of both virus and human hosts. Overall, determinants of influenza seasonality can be a mixture of viral stability, host behaviors and biology, living environments, and socioeconomic elements (41).

There are some limitations in this study. Although its focus was to assess impacts of weather variability on influenza, there might be other environmental factors that could be associated and should be considered. For instance, some studies (42, 43) indicated that absolute humidity as a humidity index is a better predictor than RH for influenza seasonality. However, we avoided using absolute humidity because of its high correlation with temperature and rainfall. Exposures to air pollution are other potentially significant indicators. This is because there is mounting evidence that home use of biomass fuel and exposure to outdoor air pollutants, such as nitrogen dioxide, particulate matter and ozone, increase the risk of respiratory viral infection (44). These other potentially significant factors should be explored in the future studies.

Despite these shortcomings, the strength of this study is to demonstrate by comprehensive study how weather variability relates to influenza in the tropics. Additionally, this is the first in-depth epidemiological study to evaluate the impacts of weather factors on disease burdens on the urban poor. Interestingly, we found from the rain effect that the unique characteristics of routine overcrowding, population demographics, and geographical locations of urban low-income communities may cause different effects of weather variability than populations of higher economic status. Moreover, there was an advantage in targeting a low-income population, which has less variation in living conditions; this is usually necessary to consider as a possible bias. The impact of air conditioner use is commonly raised as an issue regarding the effect of lowering temperature and humidity in studies of economically developed regions like Hong Kong (29). In this study, however, it was reasonable to assume a low prevalence of such high-cost electrical equipment, because many live below the poverty line with limited access to electricity.

It appears that uncertainty about the mechanisms of influenza seasonality remains. Further investigations regarding weather variability are required for better understanding, because these will also be very important from the perspective of climate change. We do not know if or how that global phenomenon will affect influenza, but a recent study reported that influenza activity has shifted to different seasons in recent years, suggesting climate change as a plausible contributor to the shift (29). Of course, urban poverty also should be integrated in the scope of investigation. Empirical evidence suggests that the proportion of urban poor is growing faster than that of the urban population itself (45). The increasing number of urban poor will face more challenges and serious public health burdens from climate change. The Intergovernmental Panel on Climate Change reports that climate change and urbanization may interact to increase health burdens and unpredicted effects (46). Enhancing our knowledge of the mechanisms of associations between seasonal influenza and weather variability for the urban poor is necessary, not only to improve ongoing intervention programs but also to protect the increasing number of the vulnerable population from the potential threats of climate change.
